# The first case of the 2015 Korean Middle East Respiratory Syndrome outbreak

**DOI:** 10.4178/epih/e2015049

**Published:** 2015-11-14

**Authors:** Yong-Shik Park, Changhwan Lee, Kyung Min Kim, Seung Woo Kim, Keon-Joo Lee, Jungmo Ahn, Moran Ki

**Affiliations:** 1 Division of Epidemic Intelligence Service, Korea Centers for Disease Control and Prevention, Cheongju, Korea; 2 Division of Public Health and Hygiene, Seoul City Hall, Seoul, Korea; 3 Department of Cancer Control and Policy, Graduate School of Cancer Science and Policy, National Cancer Center, Goyang, Korea

**Keywords:** Middle East Respiratory Syndrome, Epidemiologic studies, Transmission of infectious disease, Outbreaks, Korea

## Abstract

This study reviewed problems in the prevention of outbreak and spread of Middle East Respiratory Syndrome (MERS) and aimed to provide assistance in establishing policies to prevent and manage future outbreaks of novel infectious diseases of foreign origin via in-depth epidemiological investigation of the patient who initiated the MERS outbreak in Korea, 2015.

Personal and phone interviews were conducted with the patient and his guardians, and his activities in Saudi Arabia were investigated with the help of the Saudi Arabian Ministry of Health. Clinical courses and test results were confirmed from the medical records.

The patient visited 4 medical facilities and contacted 742 people between May 11, 2015, at symptom onset, and May 20, at admission to the National Medical Center; 28 people were infected and diagnosed with MERS thereafter.

Valuable lessons learned included: (1) epidemiological knowledge on the MERS transmission pattern and medical knowledge on its clinical course; (2) improvement of epidemiological investigative methods via closed-circuit television, global positioning system tracking, and review of Health Insurance Review and Assessment Service records; (3) problems revealed in the existing preventive techniques, including early determination of the various people contacted; (4) experiences with preventive methods used for the first time in Korea, including cohort quarantine; (5) reconsideration of the management systems for infectious disease outbreaks across the country, such as this case, at the levels of central government, local government, and the public; (6) reconsideration of hospital infectious disease management systems, culture involving patient visitation, and emergency room environments.

## INTRODUCTION

After landing in Korea in May of 2015, Middle East Respiratory Syndrome (MERS) resulted in a total of 186 confirmed cases and 36 deaths from May 11, when the symptoms occurred in the first patient, to July 4, when the last patient developed the symptoms [1]. A novel coronavirus confirmed for the first time in 2012 is suspected to be the cause of MERS and camels may be the host. Human-to-human transmission is known to occur after transmission from a camel to a human, but the exact route has not been sufficiently investigated [2]. The outbreak in Korea started with a patient who arrived in May 2015 from Saudi Arabia, a country that had an outbreak of a large number of MERS cases, and subsequently spread. A majority of infected patients were revealed to be cases of hospital-acquired infection [2,3].

This report is an epidemiological study of the first patient (Patient #1) who initially brought MERS into Korea in 2015. From May 20, when a diagnosis of MERS was confirmed, the Division of Epidemic Intelligence Service, Korea Centers for Disease Control and Prevention (KCDC), in cooperation with a group of civilian volunteers in epidemiology, traced the infection route and performed preventive measures for the spread of additional infections.

## MATERIALS AND METHODS

Through personal and phone interviews we contacted employees at business facility in Saudi Arabia who may have had contact with Patient #1 during the incubation period; we investigated the places he visited, presence or absence of MERS symptoms in the individuals he contacted, history of visiting medical facilities in the Middle East, and history of consuming camel milk or meat, among other things. The patient’s specific activities in Saudi Arabia were verified with the help of the Saudi Arabian Ministry of Health. Additionally, the timing of symptom occurrence and the initial symptoms were reevaluated by confirming the history of visiting domestic medical facilities after arrival in Korea via personal interviews with the patient and his guardians and examining his medical records. The KCDC performed a diagnostic serum antibody test for MERS for individuals whose contact with Patient #1 was confirmed by personal interviews or closed-circuit television (CCTV) reviews.

This study was conducted as an epidemiological investigation of the MERS outbreak, and thus, additional processes for review and approval by institutional review board were not required on the basis of the Life Ethics and Safety Law Enforcement Rule Item 2 (human subject studies).

## RESULTS

### Clinical course of Patient #1

**Figure 1.**
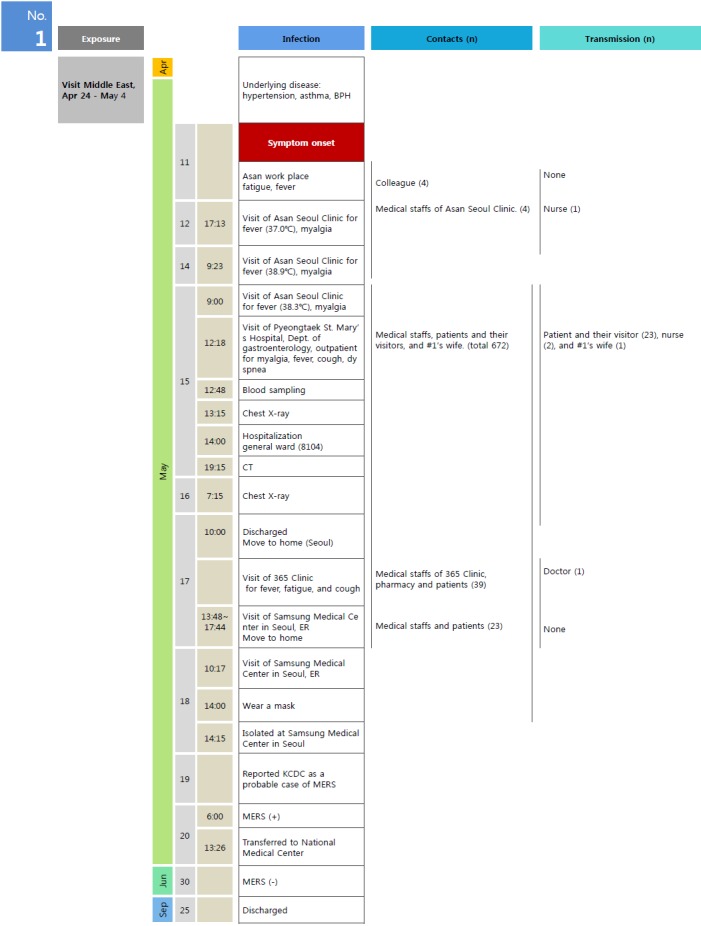
Main events by date for the 1st confirmed Middle East Respiratory Syndrome (MERS) case in Korea. BPH, benign prostatic hypertrophy; CT, computed tomography; ER, emergency room; KCDC, Korea Centers for Disease Control and Prevention.

Patient #1 was a 68-year-old man and had underlying diseases including asthma, hypertension, dyslipidemia, and benign prostate hypertrophy. At the time of the study, he was a current smoker. He was in the greenhouse building business with a business facility in the Middle East, specifically Bahrain, as well as at a domestic business facility in Asan, Chungnam, Korea. He visited the Middle East region about once every 2 months and stayed for approximately 3 weeks during each visit. The most recent business trip was between April 24, and May 4, 2015, for 11 days. He had business visits to Saudi Arabia (May 1 to 2) and the United Arab Emirates (April 29 to 30) with Bahrain as the base; both departure from and arrival at Korea was via Qatar. During his stay in the Middle East, he had no history of direct contact with camels, consuming camel by products such as milk or meat, or visiting the medical facilities there. In Saudi Arabia, he stayed at his business facility and the hotel in the area of Al Muzahimiyah outside of the capital, Riyadh. During his trips within the Middle East, he had no contact with animals and did not eat or drink outside the hotels where he stayed. While staying in Riyadh, he travelled with a driver, a guide, and the Bahrain business facility manager, none of whom showed the symptoms suspected of MERS. Patient #1 returned to Korea without any abnormal symptoms on May 4 via Flight OZ6888 and went to his domestic business place in Asan on May 11. On May 11, fever broke for the first time, and he visited Asan Seoul Clinic the next day (May 12). At the time of the visit, his body temperature was 37.0°C, and he complained of febrile sense and myalgia. On May 14, he visited the same place with a persistent high temperature (38.9°C) and myalgia, and on May 15, he was transferred to Pyeongtaek St. Mary’s Hospital for inpatient treatment, because respiratory symptoms such as cough and sputum had developed, his body temperature was 38.3°C, and the myalgia had worsened. After the transfer, he was in an outpatient exam room, a laboratory room, and a chest radiography room, and was put in a double-occupancy room at approximately 2 pm (Room 8104, 8th floor). Around 7:15 pm, a chest computed tomography (CT) scan was performed on the first underground floor. On the following day (May 16) at approximately 7:15 am, he visited the radiography room on the first underground floor to undergo a chest radiogram. Pneumonia in the right upper lobe was found on the chest CT scan, and infections with *Haemophilus influenzae* and *Streptococcus pneumonia* were confirmed on bacterial culture testing. From the evening of May 15, breathing difficulty and chest pain developed. Symptoms did not improve, and the patient was discharged from the hospital at 10 am on Sunday, May 17; thereafter, his wife drove him for treatment to the 365 Clinic, where he usually went for examination. The 365 Clinic referred him to a high-level medical center. He went to the Samsung Medical Center in Seoul emergency department, but returned home because a patient room was not available. On the following day (May 18) at approximately 10 am, his wife drove him again to the Samsung Medical Center in Seoul emergency department, and he was admitted. From 2 pm, he wore a facial mask. On May 19, he was reported to the KCDC as a case suspected of MERS in consideration of the clinical courses that deteriorated despite the antibiotic therapy and his history of travel to the Middle East within the previous two weeks, which was revealed during a consultation with physician. On May 20, his sputum polymerase chain reaction (PCR) test was determined positive, following which he was transferred to the National Medical Center. Patient #1 received antiviral, interferon, and antibiotic treatments, and mechanical ventilation therapy after an endotracheal intubation was performed due to worsening respiratory symptoms. On June 30, a sputum PCR test was confirmed negative for MERS virus, and it was decided that he was cured. On September 25, he was discharged after completing treatment for a sacral sore (Figure1).

### Individuals who made contact with Patient #1 and preventive efforts for them

Individuals who were in direct contact with Patient #1 without appropriate personal protective equipment or who stayed with him in an enclosed space (patient room, office, etc.) were considered cases of close contact and were put under surveillance. The scope of close contact surveillance gradually expanded with the spread of the MERS outbreak, and the final count of individuals under close contact surveillance was 742: 4 employees at Patient #1’s domestic business facility in Asan, 4 medical staff members at the Asan Seoul Clinic, 672 medical staff members and patients (including Patient #1’s wife) at Pyeongtaek St. Mary’s Hospital, 39 medical staff members and patients at the 365 Clinic, and 23 medical staff members and patients at the Samsung Medical Center in Seoul. Depending on the extent of contact, they were classified into either 1) quarantine of a person or a place or 2) active observational surveillance and were closely tracked and observed (Figure 1). During surveillance, when symptoms suspected as MERS occurred, respiratory samples were collected for testing, the suspected cases were transferred to a quarantined hospital, and epidemiological investigations were conducted.

### Infection transmission by Patient #1

A total of 742 people had contact with Patient #1 from May 11, when the first symptoms occurred, to May 20, when he was transferred to the National Medical Center. Of those, 28 became infected, with an infection rate of 3.8%. According to the characteristics of the contacted individuals, 4 out of 235 medical staff members (1.7%); 11 out of 206 patients (5.3%); and 13 out of 301 guardians, caregivers, and visitors (4.3%) were infected. However, trying to correlate Patient #1’s transmission capacity or epidemiological characteristics to the infection rate had limitations because with the spread of MERS, the scope of surveillance of contacted individuals gradually expanded, and the standards were not consistent across different institutions. Nonetheless, based on a study analyzing epidemiological characteristics of the MERS outbreak around Pyeongtaek St. Mary’s Hospital, the rate of infection was 3.9% overall and was 1.1% among medical staff members; 7.6% among patients; and 3.3% among guardians, caregivers, and visitors [4]. Aside from Patient #1, 3 of 28 individuals, in particular, with a confirmed diagnosis (#14, #15, and #16) individually infected more than 5 individuals whose diagnosis was additionally confirmed, and they were thus labeled as “super spreaders.” It is thought that, after contact with Patient #1, they visited different medical facilities and became epidemiologically associated with additional patients with a confirmed diagnosis [5].

## DISCUSSION

The 2015 MERS outbreak in Korea started with the infection of a businessman in his 60s who often traveled to the Middle East for business. He did not show symptoms at his arrival in Korea. A week later, he started experiencing symptoms and visited medical facilities. However, it took 9 days for the MERS infection to be confirmed at the 4th medical facility he visited, and, by then, the infection transmission had already begun at the previous 3 medical facilities. Thereafter, a “MERS crisis” transpired for the first time wherein a total of 186 patients were confirmed with MERS infection; 36 deaths occurred among these patients, and 16,693 individuals were quarantined for prevention. The clinical symptoms in the first patient deteriorated because of a delay in receiving appropriate treatment, and he was assisted with mechanical ventilation. On day 42 after admission to the 4th hospital, tests for MERS virus were negative, and the patient’s condition recovered.

From the epidemiological investigation, the course of transmission to this patient in the Middle East was not clear. It can only be inferred that the first patient became infected while visiting Saudi Arabia, on the basis of a recent study showing that the MERS virus that broke out in Korea is genetically closest to the Qatar strain [5,6]. However, to uncover the specific course of transmission, we must perform an epidemiological investigation in coordination with Saudi Arabia. Various factors are considered as causes of the widespread outbreak. The first is health care workers’ lack of knowledge regarding MERS. Since June 2013, the KCDC had organized a national MERS management team in preparation of a MERS outbreak in Korea, and mock-up training had been performed to enhance the capacity to manage outbreaks of novel infectious diseases at quarantine stations and at the level of local government of cities and provinces. However, in cases such as the present case, wherein the symptoms do not manifest themselves when patients enter Korea, preventing an outbreak of a novel infectious disease by imposing quarantine is not practical. In particular, health care workers did not receive sufficient education or communication against MERS in anticipation of a case wherein a MERS-infected individual, on whom quarantine was not imposed, would go to a regional medical facility. Until the diagnosis was confirmed for Patient #1, the first 3 medical facilities that he visited were unable to suspect a MERS case. The doctor at the 3rd medical facility, who examined Patient #1 and subsequently became infected, in an interview with the media after his discharge stated that he had not even heard of MERS. The second factor communication about MERS among travelers to the Middle East was insufficient. Since 2013, when the likelihood of spread of MERS would spread within the Middle East region was indicated, the KCDC persistently promoted MERS awareness among the travelers to that region. However, Patient #1, who frequently visited the region and his family members, had no idea about MERS. Thus, when he visited the general medical facilities with respiratory symptoms, he did not mention about his travel to the Middle East, and only mentioned about his visit to Bahrain when inquired about a travel history involving the Middle East at the medical center where his diagnosis was confirmed. Therefore, MERS could not be diagnosed sooner. A third factor contributing to the rationale behind the outbreak was not kept in check earlier was that quarantine was not thoroughly imposed. After Patient #1’s diagnosis was confirmed, thorough quarantine was neither imposed on individuals who had close contact with him nor on those with casual contact (such as those who had a possibly contacted the doctors or the patients or those who were exposed to a space infected by him). Lastly, in this outbreak, most patients were infected via hospital-acquired infection because Korean medical institutions’ patient rooms and emergency rooms were very crowded, and infected patients had to visit several hospitals because of the shortcomings of the medical care delivery system, which played a critical role in the spread of MERS from hospital to hospital.

Through an investigation on the MERS outbreak within Korea, valuable lessons were learned. They include (1) accumulation of epidemiological knowledge of the pattern of MERS transmission as well as medical knowledge on its clinical courses; (2) improvement of epidemiological investigative techniques using CCTV, global positioning system tracking, and review of Health Insurance Review and Assessment Service records, among other things; (3) problems revealed in the existing preventive techniques, including the early determination of the scope of contacted people; (4) accumulation of experiences with the preventive techniques used for the first time in Korea, such as cohort quarantine; (5) reconsideration of the management systems that deal with an outbreak of infectious diseases across the country, such as in the present case, at the levels of central government, local government, and the public; and (6) reconsideration of infectious disease management system at hospitals, culture involving patient visitation, and emergency room environments.

The 2015 MERS outbreak in Korea began with an infected businessman in his 60s who visited the Middle East region. However, the spread of outbreak is attributed to insufficient preparation and management by the Ministry of Health and Welfare. To prepare for an outbreak of novel infectious diseases in future, government organizations responsible for infectious diseases need to train personnel specialized in the area, and also promote awareness among medical staff at the primary and secondary medical facilities as well as among the public. The MERS outbreak should serve as an opportunity to improve the level of managing hospital-acquired infection in Korea.
