# Supplementary material for: The first case of the 2015 Korean Middle East Respiratory Syndrome outbreak
Source: Epidemiol Health. 2015 Nov 14;37:e2015049. doi: 10.4178/epih/e2015049 (PMC4722220; doi:10.4178/epih/e2015049)
Supplement: Supplementary file 1 [file epih-37-e2015049-supplementary.pdf]

2015 한국 MERS 유행의 발단환자에 대한 역학조사 보고

박용식[1], 이창환[1], 김경민[1], 김승우[1], 이건주[1], 안정모[2], 기모란[3]

[1]질병관리본부 역학조사과, 청주

2서울시청 생활보건과, 서울

[3]국립암센터 국제암대학원대학교 암관리정책학과, 고양

교신저자: 기모란

The first case of 2015 Korean MERS outbreak

Yong-shik Park[1], Changhwan Lee[1], Kyung Min Kim[1], Seung Woo Kim[1], Keon-Joo Lee[1], Jungmo Ahn[2], Moran Ki[3]

[1]Division of Epidemic Intelligence Service, Korea Centers for Disease Control and Prevention, Cheongju

[2]Division of Public health and hygiene, Seoul city hall, Seoul

[3]Department of Cancer Control and Policy, Graduate School of Cancer Science and Policy, National Cancer Center, Goyang

ORCID

Yong-Shik Park, http://orcid.org/

Changhwan Lee, http://orcid.org/ 0000-0001-9574-4333

Kyung Min Kim, http://orcid.org/ 0000-0002-0261-1687

Seung Woo Kim, http://orcid.org/

Keon-Joo Lee, http://orcid.org/

Jungmo Ahn, http://orcid.org/

Moran Ki, http://orcid.org/ 0000-0002-8892-7104

Number of references: 6

This article includes 1 Figure

Running title: The first case of 2015 Korean MERS outbreak

Corresponding author: Moran Ki (moranki@naver.com)

<초록>

목적: 2015년 한국 메르스 유행의 발단이 된 첫 번째 환자의 심층역학조사를 통해 메르스 유입 및 유행 확산을 막기 위한 대응 과정의 문제점을 되짚어 보고, 향후 신종감염병의 국내유입을 막기 위한 대비•대응 정책 수립에 도움이 되고자 한다.

방법: 역학적 내용은 환자 및 보호자와의 직접 또는 전화 면접조사, 사우디아라비아 보건부와의 협조 하에 현지 활동내역을 조사하였고, 임상 경과 및 검사 결과는 의무기록을 바탕으로 확인하였다.

결과: 첫 번째 환자가 증상이 발생한 2015년 5월 11일부터 국립중앙의료원에 입원하는 5월 20일까지 총 4개 의료기관을 경유하면서 735명과 접촉하였고, 이 과정에서 28명의 메르스 확진자가 발생하였다.

결론: 이번 유행 확산의 가장 큰 원인으로는 1. 의료기관을 포함한 메르스에 대한 전반적 인식 부재, 2. 메르스 유행 초기에 적절한 접촉자 관리가 이뤄지지 않은 점, 3. 한국 특유의 병문안 문화와 응급실 환경이 빚은 의료기관 내 인구 과밀화, 4. 미숙한 원내감염 관리를 들 수 있다. 이러한 점들이 반영되어 신종감염병 대비•대응 방침이 수정•보완되어야 할 것이다.

.

<주제어>

MERS

Epidemiologic investigation

Mode of transmission

Outbreak

Korea

서론(Introduction)

2015년 5월 한국에 유입된 중동호흡기증후군(이하 메르스)은 첫 환자의 증상 발생일인 5월 11일 이후 마지막 환자의 증상발생일인 7월 4일까지 총 186명의 확진자와 36명의 사망자를 낳았다[1]. 메르스는 2012년에 처음 확인된 변형 코로나바이러스가 그 원인으로, 낙타가 숙주로 의심되고 있으며 낙타에서 사람으로 감염된 후 사람 간 감염이 일어날 수 있는 것으로 알려져 있지만 정확한 경로는 충분히 연구되어있지 않다[2]. 2015년 5월 호발 국가인 사우디아라비아에서 한국에 처음으로 환자가 유입되어 발생한 유행에서, 대부분의 감염자들은 원내감염인 것으로 밝혀지고 있다[2, 3].

이 조사는 2015년 한국 메르스 유행의 발단이 된 첫 번째 환자(이후 #1 환자)에 대한 역학조사로서, 환자가 확진 된 5월 20일부터 질병관리본부 역학조사반과 민간역학조사지원단이 협력하여 감염경로를 추적하고 추가 감염 확산을 방지하기 위한 방역조치를 시행하였다.

방법(Methods)

잠복기 동안 #1 환자와 접촉가능성이 있는 중동 현지 사업장의 직원을 대상으로 직접 및 전화 면접조사를 통해 중동지역 체류 시 방문지역, 접촉자 중 메르스 증상자 유무, 현지 의료기관 방문력, 낙타유·고기 섭취력 등을 조사하였다. 또한 사우디아라비아 보건부의 협조 하에 사우디아라비아 현지에서의 구체적인 활동 내역을 확인하였다. 이외에 환자 및 보호자와의 직접 면접조사를 통해 국내 입국 이후 의료기관 방문력을 확인하고 의무기록을 검토하여 증상 발생 시점과 초기 증상을 재평가하였다. 직접 면접조사 및 CCTV 검토를 통해 확인된 #1 환자와의 접촉자들을 대상으로 메르스 진단을 위한 혈청 항체검사를 질병관리본부에서 실시하였다.

메르스 유행 역학조사로 수행되었기 때문에 생명윤리 및 안전에 관한 법률 시행규칙 제 2조(인간대상연구의 범위)에 의거하여 별도의 기관윤리위원회 심사 및 승인절차는 필요하지 않았다.

결과(Results)

(#1환자의 임상 경과)

#1 환자는 68세 남자로 천식, 고혈압, 고지혈증, 전립선 비대증의 기저질환이 있으며 현재 흡연자이

다. 온실 건설관련 사업으로 바레인에 중동 현지 사업장이 있으며 충남 아산에 국내 사업장이 있어 2개월에 한 번 정도 중동지역을 방문하고, 방문 시 3주 정도 머물렀다고 한다. 가장 최근 출장은 2015년 4월 24일부터 5월 4일까지 11일 동안이었으며, 바레인을 거점으로 사우디아라비아(5월 1일 -2일)와 아랍에미리트(4월 29일-30일)를 사업 차 방문하였고, 카타르를 거쳐 입·출국하였다. 중동지역에 머무르는 동안 낙타와의 직접 접촉력이나 낙타유·고기와 같은 낙타 부산물 섭취력은 없었으며, 현지 의료기관 방문력 또한 없었다. 사우디아라비아에서는 수도 Riyadh 외곽의 Al Muzahimiyah 지역 내 사업장과 호텔에 체류하였고, 이동간 동물 접촉은 없었으며 숙소 외부에서 식음료 섭취는 없었다. Riyadh 체류 시 운전기사, 가이드, 현지 사업장 감독관 등 3명과 동행하였지만 메르스 의심 증상을 보인 사람은 없었다. #1 환자는 별다른 이상증상 없이 5월 4일 OZ6888편으로 귀국하였고 5월11일 충남 아산의 국내 사업장으로 갔다. 5월 11일 발열이 최초 발생하여 다음날인 5월 12일 아산에 있는 서울의원을 방문하였다. 내원 당시 체온은 37.0℃였고 열감과 근육통을 호소하였다. 5월 14일에도 38.9℃ 발열과 근육통이 지속되어 동일 의료기관을 방문하였고, 5월 15일에 기침, 가래와 같은 호흡기 증상이 발현되면서 38.3℃의 발열 및 근육통이 악화되어 입원치료 위해 평택성모병원으로 전원 되었다. 전원 후 외래 진료실, 채혈실, 흉부 방사선 촬영실을 경유하여 오후 2시경 2인실(8층 8104호)에 입원하였으며, 오후 7시 15분경에 지하 1층 흉부 CT 촬영을 시행하였다. 다음날인 5월 16일 오전 7시 15분경 흉부 엑스레이 촬영 위해 지하 1층 방사선 촬영실을 방문하였다. 흉부 CT 소견에서 우상엽 폐렴 소견이 관찰되었으며 세균 배양검사에서 Haemophilus influenza, Streptococcal pneumonia가 동반 감염된 것으로 확인되었다. 5월 15일 저녁부터 호흡곤란 및 흉부통증 증상이 발생하였고 이후 증상이 호전 되지 않아 5월 17일 일요일 오전 10시에 퇴원하고, 부인과 함께 자차로 평소 진료를 받아오던 서울 천호동 365 연합의원에서 진료를 받았다. 이곳에서 상급 의료기관 방문을 권유 받아 삼성서울병원 응급실에 갔으나 입원실이 없어 자택으로 돌아와 하룻밤을 지내고 다음날인 5월 18일 오전 10시경에 부인과 자차로 삼성서울병원 응급실을 다시 방문하여 입원하였다. 오후 2시경부터는 마스크를 착용하였고 항생제 치료에도 불구하고 악화되는 임상 경과와 주치의의 병력청취 과정에서 청취된 최근 2주 이내 중동지역 여행력을 고려하여 5월 19일 질병관리본부에 메르스 의심 사례로 신고되었으며, 5월 20일 객담 PCR 검사에서 양성으로 판정되어

국립중앙의료원으로 전원 되었다. #1 환자는 항바이러스제와 인터페론 그리고 항생제 치료를 받았으며, 호흡기 증상 악화로 기관삽관 후 기계호흡 치료를 병행하였다. 6월 30일 객담 PCR 검사에서 메르스 바이러스 음성소견이 확인되어 완치 되었다고 판단되었으며, 10월 1일 현재 천추부 욕창 치료를 위해 입원 치료 중이다. (그림 1).

(#1 환자의 접촉자 및 이들에 대한 방역)

#1 환자와 적절한 개인보호장비 없이 직접적 접촉이 있었거나, 제한된 공간(병실, 사무실 등) 내에서 머무른 경우 밀접 접촉으로 간주하여 관리하였다. 접촉자 관리 대상은 메르스 유행 확산에 따라 조금씩 증가하였는데 최종 관리대상자는 735명으로 아산 국내 사업장 직원 4명, 아산 서울의원 의료진 4명, 서울 365 연합의원 의료진 및 환자 37명, 평택성모병원 의료진 및 환자 673명(#1 환자의 부인 포함), 삼성서울병원 의료진 및 환자 17명이 해당되었으며, 이들을 접촉 강도에 따라 1)자가격리 또는 시설격리와 2)능동감시 대상자로 분류하여 추적 관찰하였다 (그림 1). 관찰 기간 중 메르스 의심 증상 발생시 호흡기 검체 채취 및 격리병원 이송과 역학조사를 실시하였다.

(#1 환자에 의한 감염 전파)

#1 환자의 접촉자 중 28명에게서 메르스가 확진 되었다. 여기에는 아산 서울의원 의료진(간호사) 1명, 서울 365 연합의원 의료진(의사) 1명이 해당 되었고, 평택성모병원에서는 의료진(간호사 2명)과 입원 중이던 환자(14명)와 보호자(9명), #1 환자의 부인(1명)을 합하여 총 28명이 메르스로 확진 되었다[4].

28명의 확진자중, 특히 #14, #15, #16, #76의 4명의 확진자는 #1 환자와 더불어 각각 5명 이상의 추가 확진자를 발생시킨 것으로 확인되어 ‘슈퍼전파자(Superspreader)’로 분류되었으며, 이후 다른 의료기관을 방문하여 추가 확진자의 83.2%인 153명과 역학적으로 연관이 있는 것으로 생각된다.[5]

토의(Discussion)

2015년 한국의 메르스 유행은 중동을 오가며 사업을 하던 한 60대 남자의 감염으로 시작되었다. 한

국에 입국할 당시에는 증상이 없었고, 입국하고 일주일 후 증상이 발생하여 의료기관을 찾았지만 4번째 방문 의료기관에서 메르스로 확진 될 때까지 9일이 걸렸고, 이때는 이미 3곳의 의료기관에서 감염전파가 이루어진 다음이었다. 이후 총 186명의 확진자가 나왔고, 이중에 36명이 사망하였으며, 방역을 위하여 16693명에 대하여 격리조치가 취해지는 등 초유의 “메르스 위기”가 발생하였다. 발단 환자는 적절한 치료가 늦어지면서 임상 증상이 악화되어 인공호흡기 치료를 받았으며, 입원 42일째에 메르스 바이러스는 음성으로 전환되었고, 환자의 상태도 회복되었다.

역학조사결과 이 발단환자의 중동지역 내에서의 감염 경로는 분명하지 않았다. 다만 최근의 논문에 의하면 이번에 한국에서 유행한 메르스 바이러스는 유전적으로 Qatar strain 과 가장 가까운 것으로 나타나서 발단환자가 사우디아라비아를 방문하였을 때 감염된 것으로 추정된다[5,6]. 하지만 자세한 감염 경로를 밝히기 위해서는 사우디아라비아와의 협동 역학조사가 필요하다. 이후 유행이 확산된 원인은 여러 가지 요인을 고려해 볼 수 있다.

첫 번째는 일선 의료기관에서 메르스에 대한 지식이 부족한 것이었다. 질병관리본부는 2013년 6월부터 메르스 국내 유입에 대비하여 중앙 메르스 대책반을 운영하였으며, 검역소 및 시·도 지방자치단체 차원에서 메르스와 같은 신종감염병의 유입 시 대응 역량 강화를 위해 모의 훈련을 수행해 왔다. 하지만 이번 사례에서와 같이 국내 입국 당시에 증상발현이 되지 않는 경우 검역차원에서 신종감염병의 유입을 막기에는 무리가 있었다. 특히, 검역에서 걸러지지 않은 메르스 감염자가 지역의 의료기관에 갈 경우를 대비한 일선 의료진에 대한 메르스 교육이나 홍보가 충분하지 못했다. #1 환자가 확진 될 때까지 거쳐간 3개의 의료기관에서는 메르스에 대하여 의심조차 하지 못하였다. #1 환자가 세 번째 방문하였던 의원의 의사도 메르스에 감염이 되었는데 퇴원 이후에 가진 언론 인터뷰에서 이전에 메르스에 대하여 전혀 들어본 적이 없다고 하였다. 두 번째 요인은 중동지역 여행객에게도 메르스에 대한 홍보를 충분히 하지 못한 것이었다. 질병관리본부는 중동지역 내 메르스 확산징후를 보인 2013년부터 중동지역 여행객들을 대상으로 메르스 홍보를 지속적으로 해왔으나 중동지역을 빈번히 오가는 #1 환자나 그 가족은 메르스에 대하여 전혀 알지 못했다. 이로 인해 호흡기 증상으로 일선 의료기관을 찾았을 때 중동 여행력을 말하지 않았고, 확진을 받았던 의료기관에서도 중동 여행력에 대한 질문에 바레인을 다녀온 사실만 이야기 하였다. 이는 메르스를 보다 빨리

진단하지 못하게 된 원인이 되었다. 세 번째는 #1 환자가 확진 된 이후 #1 환자와 밀접하게 접촉한 밀접 접촉자 뿐만 아니라 일상적 접촉자(의사환자와 접촉할 가능성이 있거나, 확진 환자에 의해 오염된 공간에 노출된 사람)를 대상으로 철저하게 방역을 시행하지 못한 것이 초기 유행 확산을 막지 못한 요인이 되었다. 마지막으로 이번 유행은 대부분의 환자가 병원감염으로 인하여 감염이 되었는데 이는 우리나라 의료기관의 특성상 병실과 응급실이 매우 혼잡하고, 부실한 의료전달체계로 감염 환자들이 여러 병원을 거쳐가면서 병원에서 병원으로 질병을 전파시킨 것이 유행 확산에 결정적 역할을 하였다.

본 국내 메르스 유행을 통해 (1) 메르스의 전파양상에 대한 역학적 지식 및 임상경과에 대한의학적 지식 축적, (2) CCTV, GPS 추적, 건강보험심사평가원 기록 조회 등을 통한 역학조사 기법 향상, (3) 초기 접촉자 범위 설정 등 기존 방역 기법에 있어서의 문제점 도출, (4) 코호트 격리 등 국내에서 처음으로 적용된 방역 기법에 대한 경험 축적, (5) 본 유행과 같은 범국가적인 감염병 유행 시의 정부, 지자체, 민간차원에서의 대응 시스템에 대한 재고, (6) 병원 감염관리시스템과 간병문화, 응급실 환경에 대한 재고와 같은 귀중한 교훈들이 있었다.

결론 (Conclusion)

2015년 한국 메르스 유행은 60대 중동지역을 방문하였던 한 사업가의 감염으로 시작되었다. 하지만 유행의 확산은 보건 당국의 대비 대응이 부족했던 것에서 기인 되었다. 앞으로 또다시 닥쳐 올 수 있는 신종감염병의 유행에 대비하기 위해서는 감염병을 책임지는 정부 기관에서 전문인력의 양성과 함께 일선 1, 2차 의료기관의 의료진과 일반 시민들에게도 충분한 홍보를 하여 대비할 필요가 있다. 이번 메르스 유행이 한국의 병원감염 관리 수준이 향상되는 계기가 되어야 할 것이다.

감사의 글(Acknowledgements)

참고문헌(References)

1. Moran Ki. 2015 MERS outbreak in Korea: hospital-to-hospital transmission. Epidemiol Health. 2015;37:e2015033 Published online 2015 July 21.

2. Zumla A, Hui DS, Perman S. Middle East respiratory syndrome. Lancet. 2015 Jun 3. pii: S0140-6736(15)60454-8. doi: 10.1016/S0140-6736(15)60454-8. [Epub ahead of print]

3. Choi JW, Kim KH, Cho YM, Kim SH. Current epidemiological situation of Middle East respiratory syndrome coronavirus clusters and implications for public health response in South Korea. J Korean Med Assoc. 2015 June; 58(6): 487-497

4. KM Kim et al. Epidemiologic features of the first MERS outbreak: Pyeongtaek St.Mary's Hospital, Korea(on submission). Epidelmiol Health. 2015

5. KCDC MERS-CoV Outbreak Investigation Team. MERS-CoV outbreak in the Republic of Korea, 2015 (accepted). PHRP. 2015

6. Durai P, Batool M, Shah M, Choi S. Middle East respiratory syndrome coronavirus: transmission, virology and therapeutic targeting to aid in outbreak control. Exp Mol Med. 2015 Aug 28:47:e181.
